# A web-based prospective cohort study of home, leisure, school and sports injuries in France: a descriptive analysis

**DOI:** 10.1186/s40621-021-00343-9

**Published:** 2021-08-04

**Authors:** Madelyn Yiseth Rojas Castro, Ludivine Orriols, Dunia Basha Sakr, Benjamin Contrand, Marion Dupuy, Marina Travanca, Catherine Sztal-Kutas, Marta Avalos, Emmanuel Lagarde

**Affiliations:** 1 University of Bordeaux, Bordeaux Population Health Research Center, UMR U1219, INSERM, Bordeaux, France; 2 Calyxis, center of risk expertise, Niort, France; 3 SISTM team Inria BSO, Talence, France

**Keywords:** Wounds and injuries, Safety, Home accident, Falls, Prospective studies

## Abstract

**Background:**

Home and leisure injuries (HLIs) are a major public health problem. Cohort studies among general population are needed for targeted preventive actions but remain scarce. We quantify and qualify the HLIs collected prospectively in the MAVIE (Mutualists against Home and Leisure Injuries) observatory, a web-based cohort among volunteers of the French general population.

**Methods:**

Participants reported HLIs from November 2014 to December 2019. We calculated crude and standardized incidence rates (SIRs) on the entire cohort, for each of the selected socio-demographic variables and each of the injury circumstances (place and activity), mechanisms, and injury severity levels. We also described other HLIs characteristics and consequences.

**Results:**

Out of the 29,931 household members enrolled in the cohort, 12,419 participants completed the questionnaires. Among them, 8640 participants provided follow-up data, leading to a follow-up of 6302 persons for 5.2 years and 2483 HLIs were reported. We obtained a SIR of 85.0 HLIs per 1000 persons-years. Most reported injuries did not require emergency department attendance or hospitalization (64%). SIRs were higher in children (< 15 years of age) (109.1 HLIs per 1000 persons-years; 95% CI, 78.2–140.1) and adults aged 70 years and older (123.7 HLIs per 1000 persons-years; 95% CI, 79.2–168.3). *Struck or hit by fall* was the most frequent injury mechanism (52%) and also among the most severe injuries (73% of *Struck or hit by fall* HLIs ending with hospitalization). Sport (without contact with nature), and *leisure and play* activities were the injury circumstances with higher SIRs, 15.2 HLIs per 1000 persons-years (95% CI, 14.6–15.8) and 11.2 HLIs per 1000 persons-years (95% CI, 10.7–11.6), respectively. Outdoor sport activity (in contact with nature) was the circumstance with the highest proportion of hospitalizations (18% of outdoor sports HLIs ending with hospitalization).

**Conclusion:**

The incidences, causes, and consequences of HLI differ by age group and are mainly related to the performance of certain activities. Although the participants in the MAVIE cohort were not representative of the French population. Our study identified potential sub-populations and specific types of HLIs that should be targeted by future studies concerning risk factors and prevention programs.

**Supplementary Information:**

The online version contains supplementary material available at 10.1186/s40621-021-00343-9.

## Background

Home Leisure Injuries (HLIs) refer to a wide range of events occurring at any time in private life (including also sport and school injuries), except injuries due to road traffic injuries, occupational injuries, suicide, violence, or aggression. HLIs are a substantial cause of mortality, morbidity and an important contributor to health costs. In 2017, according to estimates from the Global Burden of Disease Study, 415 million people suffered unintentional injuries (excluding road traffic injuries), and 1.8 million died (Roser & Ritchie, 2016). HLIs were the most frequent type of unintentional injury.

In 26 countries of the European Union (EU), there are 25 million people attending the emergency departments (EDs) every year, and 113 thousand people die due to HLIs (Eurosafe, 2016). Between 2013 and 2016, falls were the leading cause of fatal injuries (22%), followed by road traffic injuries (13%), poisonings (10%), cuts and piercings (4%), and burns and scalds (2%) (Eurosafe, 2016). The burden of injuries is unevenly distributed among populations, areas and countries and depends on the steepness of the social gradient, health status, lifestyle and local policies (Zambon & Loring, 2014; Stewart et al., 2012; Polinder et al., 2007; Smith, 1991).

Successful preventive interventions require the availability of HLIs data at all levels. Injury Surveillance Systems based on ED attendance and hospitalization data are the primary sources. In the United States, a valuable source is the National Electronic Injury Surveillance System that collects ED attendance information related to consumer products (Consumer Product Safety Commission) (U.S. Consumer Product Safety Commission, 1977). In Canada, the Canadian Hospitals Injury Reporting and Prevention Program provides information on injury circumstances in some pediatric and general hospitals (Mackenzie & Pless, 1999). In the EU, the association EuroSafe (European Association for Injury Prevention and Safety Promotion) aggregates data from member countries into the EU-Injury Database (Lyons et al., 2016). In France, the French Home and Leisure Injury Permanent Survey (*Enquête Permanente sur les Accidents de la vie Courante* - EPAC) is the primary HLIs data source. It has been in operation since 2000, with a sample of 20 thousand HLIs every year (Institut de veille sanitaire, 2005). Other HLIs sources of data are mortality registers and specific population health surveys, as the Health, Health Care and Insurance Survey (*l’Enquête Santé et Protection Sociale* – ESPS) (Paget & Thélot, 2017).

In spite of the availability of surveillance systems, preventive actions are hampered by the lack of knowledge of exposure factors and information on the characteristics of individual vulnerabilities. Longitudinal data are needed to explore the association of emerging factors. So far, studies with regard to the HLIs in the general population are scarce; existing cohort studies focus mainly on children and young adults (Zonfrillo et al., 2018), sports injuries (Van Der Worp et al., 2015), and falls in older adults (Ho et al., 2019). Finally, there are limited data on the epidemiology of HLIs and different severity levels of the injuries: hospitalization, ED attendance, or simple medical care.

In response to these challenges, we developed a cohort study in France, i.e., the Mutualists against Home and Leisure Injuries observatory (*Mutualistes pour la recherche contre les Accidents de la VIE courante* – MAVIE), intending to survey HLIs of any severity level and to elucidate the most relevant exposures and risk factors. We describe in this article the incidences, circumstances, mechanisms, consequences, and other characteristics of the injury events collected from the MAVIE cohort.

## Methods

### Aim, design and setting of the study

The MAVIE observatory is a web-based prospective cohort study, with a longitudinal follow-up of HLIs (Rojas-Castro et al., 2021). All households in France and in French overseas territories were eligible to participate. The recruitment process began in November 2014 and is still ongoing. This article analyzes the information collected up to 31 December 2019.

Cohort management was entirely online, including invitations, registration, and data collection. The largest share of participants was recruited through an email invitation sent to their insurees by three mutual insurance companies (MAAF, MACIF, and MAIF). A smaller proportion of the participants was informed of the MAVIE observatory and invited to participate through press releases, social media, posters, flyers, and material incentives.

Potential participants are asked to choose a household reference member, who receives all correspondence and reminder messages and is in charge of reporting HLIs that may happen to any consenting household members. Each member was free to participate or not and had to provide individual consent.

The inclusion criteria were: 1) residing in France, 2) being able to answer the questionnaires in French, 3) having access to and being able to use the Internet (at least the reference member). In an attempt to address the foreseeable underrepresentation of older participants who may have difficulties using computers, another participation status was created for caregivers whose only role is to represent older persons. It was also possible to participate to represent a child. We describe other attempts to reduce bias in the MAVIE observatory in elsewhere (Rojas-Castro et al., 2021).

### Data collection

Adults who signed a consent form to participate in the study by themselves or their children were asked to complete several web-based questionnaires. These inclusion questionnaires were designed to collect information including, socio-demographic characteristics, health, domestic, sport and leisure activities, lifestyles, and the home characteristics of each participants.

### Follow-up

Events could be reported at any time on the website. In addition, every three months, the reference member received a reminder email to invite him/her to report any injury events that may have happened to any participant members of the household. If no event had occurred, a link in the email made it possible to report with a single click. Injury-reporting questionnaire collects information on the circumstances of occurrence (the main activity and place for each injury event), the multiple mechanisms (such as impact, crushing and cutting), the type of medical care, the typology of injury (fracture, contusion), the injury body location, limitations on doing usual activities for the 48 h following the event and the total number of lost work/study days and. Injury variables were coded using the same format as that developed for the EU-Injury database and the EPAC survey (Thélot et al., 2004).

### Participant data

We considered as participants all the household members reported by the reference member who agreed to participate in the study. The baseline sample was defined as participants who gave an answer to at least one question in the individual inclusion questionnaire. Additionally, we defined a follow-up sample as people who reported at least one news statement to the cohort.

### HLI data

We excluded all clearly identifiable, all events involving illness, medical symptoms, and all reported events during which no injury was sustained, or that concerned someone other than the participant. In order to fulfil the definition of HLI, we excluded all road or occupational injuries that may have been mistakenly reported. Finally, all iatrogenic events were excluded. We excluded events that occurred before the entry into the study and those that were reported on the same date as the consent date. Finally, injuries were only included when information about the type of medical care, mechanism, place or activity were reported.

### Statistical analysis

Data were analyzed using R 3.6.1 (R Development Core Team, n.d.). We considered the follow-up time from the consent date to: the last news statement date, or the questionnaires update date. If the participant asked to leave the study, we used the cohort exit date or the date of death, if reported. In an attempt to evaluate the completeness of the cohort follow-up, we computed the Person-Time Follow-up Rate (PTFR), which consists of the observed person-time (until the last news statement date) divided by the total person-time assuming no dropouts (time from the consent date to the cut-off date) (Xue et al., 2017). We compared the socio-demographic characteristics between the sample of participants at baseline and the sample of participants who provided follow-up data (follow-up sample).

We assigned the reported highest education level according to the percentiles of “educational attainment by age”: low (< 50th percentile) or high (≥ 50th percentile). We considered that “low educational attainment” corresponded to bachelor’s degree or lower levels among participants aged 20–54 years, and to primary, general education or lower levels among participants of other ages. We divided household incomes into classes based on the French population in the 2015 census: low (≤ 30th percentile), medium (30th - 80th percentile) and high (≥ 80th percentile) income.

We defined injury severity levels based on the type of medical care for the reported injuries in three mutually exclusive categories: 1) Hospitalization, 2) ED attendance, 3) Neither ED nor hospitalization. The last category includes medical care, nursing care, dental care, physiotherapy, other medical care, the care provided by the victim him/herself and non-medical personnel. In addition to this, we defined a secondary outcome, the injuries requiring any medical care, including hospitalization, ED attendance, and others care services. In addition, we grouped HLIs by place and activity, looking for similar circumstances of occurrence.

We calculated Crude Incidence Rates (CIR) and Standardized Incidence Rates (SIR) on the entire cohort for socio-demographic variables and groups of injury circumstances, mechanisms and severity levels. SIRs were calculated using direct standardization on the age and sex distribution in France in 2015. CIR and SIR 95% confidence intervals were computed using the Poisson approximation (Fay & Feuer, 1997). In addition, we described the typology of injury, injury body location, limitations on doing usual activities in the 48 h following the event, and the number of work and study days lost.

## Results

### Participants

**Fig. 1:**
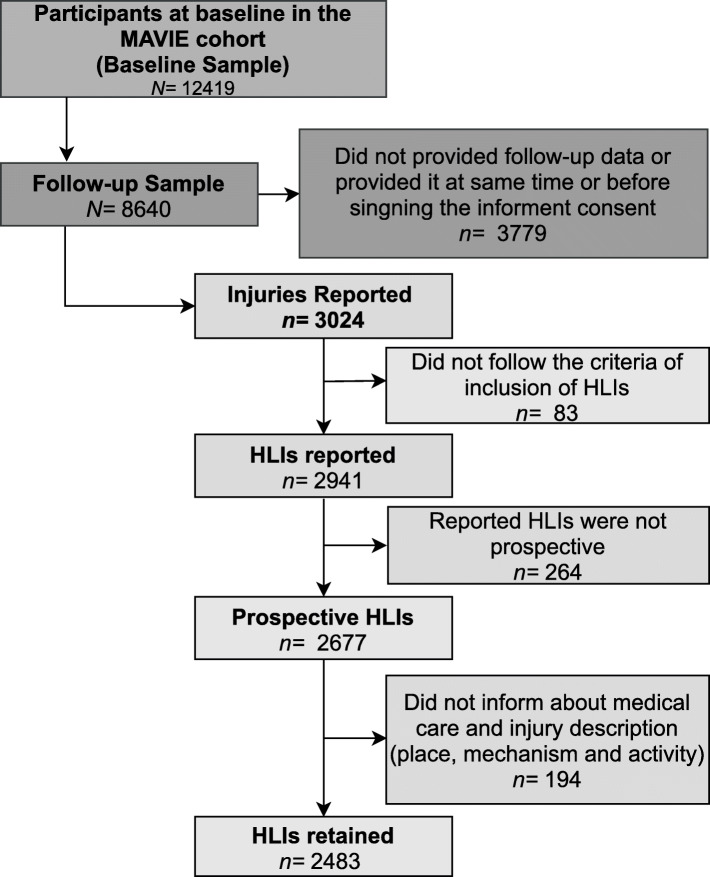
Flow diagram for the selection of the individuals and the HLI events

Between November 2014 and December 2019, 12,419 people signed a consent form to participate in the MAVIE cohort and responded to the baseline questionnaires, and 8640 provided follow-up data at least once (Fig. 1). The median and maximum follow-up time were 4.0 years (Q_1_ = 3.6, Q_3_ = 4.5) and 5.2 years, respectively. The observed person-time was 6302 persons followed for 5.2 years. The person-time assuming no dropouts was 7312 persons followed for 5.2 years when considering the whole period from recruitment to the cut-off date (31 of December 2019). Loss to follow-up, defined as one less the ratio of those estimations (1- PTFR), was 13.8%.

Among the participants in the follow-up sample, 4511 were women (52%), and 4129 were men (48%). Their mean age was 50 ± 20 years, 48 ± 20 years for women, and 52 ± 20 years for men. The maximum age was 101 years. We observed differences between the baseline and follow-up samples concerning all variables except sex (Table 1). However, these differences were not relevant comparing figures of both samples. Additional file 1 shows the percentage of practitioners of different activities (domestic, gardening, DIY, sports, outdoor and indoor play) by age and sex.

**Table 1:** Comparison of the MAVIE baseline and follow-up samples

Participants Characteristics	Baseline Sample		Follow-up Sample		***P***-value
All	***n*** = 12,419	(%)	***n*** = 8640	(%)	
**Sex**					
Male	5838	(47)	4129	(48)	0.270
Female	6581	(53)	4511	(52)	
**Age groups (years)**					
< 15	999	(8)	763	(9)	< 0.001
15–34	1795	(14)	1092	(13)	
35–54	3584	(29)	2297	(27)	
55–69	4539	(36)	3396	(39)	
≥ 70	1502	(12)	1092	(13)	
**Occupational Status (≥ 15 years old)**					
Farmer, operators, craftsman, shopkeeper	127	(1)	91	(1)	< 0.001
Higher manager, professional occupations, independent	1783	(17)	1347	(19)	
Middle manager, employees	2386	(25)	1619	(19)	
Retired	3819	(41)	3017	(43)	
Unemployed	871	(9)	615	(8)	
Student	299	(3)	192	(2)	
*Other*	312	(3)	202	(3)	
*missing*	1823		794		
**Educational attainment by age (≥ 15 years old)**					
Low	8738	(91)	6515	(92)	0.038
High	868	(9)	575	(8)	
*missing*	1819		787		
**Household Characteristics**					
**All**	**9483**		**6356**		
**Number of household members**					
1 member	2198	(23)	1563	(25)	< 0.001
2 members	4211	(44)	2953	(46)	
3 members	1308	(14)	788	(12)	
4 members	1222	(13)	756	(12)	
5 members	408	(4)	228	(4)	
6 members and more	113	(1)	53	(1)	
*missing*	23		15		
**Household income level**					
Low ≤ p30	1177	(14)	704	(13)	0.006
Medium (p30 – p80)	3319	(41)	2258	(41)	
High ≥ p80	3651	(45)	2598	(47)	
*missing*	1336		796		

### HLIs frequency and incidence rates

Participants who provided follow-up data reported 3024 events between the inclusion and the cut-off date. Among them, 83 events did not match the HLIs criteria. We excluded 264 events because they occurred before or on the inclusion date. We excluded another 194 events because data related to the type of medical care, mechanism, place, or activity were not available. The analyses included 2483 HLIs (Fig. 1). Among participants, 6942 (80%) reported no injury, while 1698 (20%) reported at least one HLI during the period. 48% reported an event among the victims, the mean number of events was 1.5 ± 1.0, and the first event was 20 ± 15 months.

**Fig. 2:**
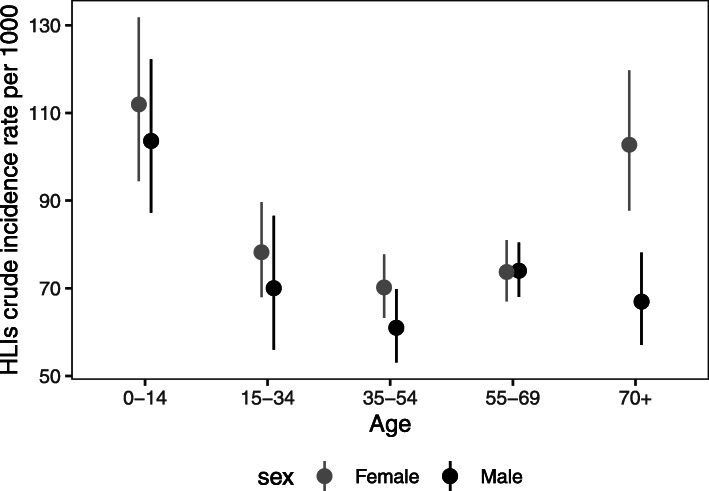
HLI crude incidence rate by age-sex group: crude incidence rate per 1000 person-years, 95% confidence intervals

With 32,770 person-years of follow-up in the MAVIE cohort, we estimated the SIR of non-fatal HLIs (with or without medical care) in the French population at 85.0 HLIs per 1000 person-years (95% CI, 60.6–109.4). The CIR of HLIs was significantly higher in women, except in the 55–69 age group. We obtained a U-shaped curve, higher CIRs in children and in participants over 70 years of age. However, the CIR of HLIs was much higher among women over 70 years of age than among men of the same age (Fig. 2). The SIRs of HLIs were higher among members of high-income households; among *higher managers, participants with professional occupations or independents* and among retired or pre-retired people; among those living alone and in four-member households (Table 2).

**Table 2:** Number of victims, events and the HLIs incidence rates in relation to demographic variables in the MAVIE follow-up sample

Participants Characteristics	Injured^**a**^	(%)	HLIs ^**b**^	CIR (95% CI) ^c^			SIR (95% CI) ^d^		
All	1698	(20)	2483	75.8	(72.8	– 78.8)	85.0	(60.6	– 109.4)
**Sex**									
Male	806	(19)	1158	72.3	(68.2	– 76.6)	75.0	(51.3	– 98.7)
Female	892	(20)	1325	79.0	(74.8	– 83.4)	94.4	(69.3	– 119.5)
**Age groups (years)**									
15	184	(24)	285	107.7	(95.5	– 120.9)	109.1	(78.2	– 140.1)
15–34	189	(17)	293	75.6	(67.2	– 84.8)	76.4	(48.2	– 104.7)
35–54	411	(18)	584	66.6	(61.3	– 72.2)	65.5	(50.1	– 80.9)
55–69	687	(20)	994	73.9	(69.4	– 78.6)	72.7	(61.2	– 84.1)
≥ 70	227	(21)	327	81.2	(72.7	– 90.6)	123.7	(79.2	– 168.3)
**Occupational Status (≥ 15 years old)**									
Farmer, operators, craftsman, shopkeeper	13	(14)	17	47.2	(27.5	– 75.6)	–	–	–
Higher manager, professional occupations or independent	293	(22)	422	80.3	(72.8	– 88.3)	–	–	–
Middle manager, employee	276	(17)	401	64.0	(57.9	– 70.5)	–	–	–
Retired	671	(22)	984	81.8	(76.8	– 87.1)	–	–	–
Unemployed	110	(18)	168	69.7	(59.5	– 81.0)	–	–	–
Student	32	(17)	48	71.6	(52.8	– 94.9)	–	–	–
*Other*	39	(19)	59	76.1	(57.9	– 98.2)	–	–	–
**Educational attainment by age (≥ 15 years old)**									
Low	91	(16)	123	(55)	(46.8	– 67.2)	–	–	–
High	1345	(21)	1978	(77)	(73.9	– 80.7)	–	–	–
**Member in a household of**									
1 member	367	(23)	587	97.0	(89.3	– 105.1)	–	–	–
2 members	770	(19)	1090	71.3	(67.2	– 75.7)	–	–	–
3 members	195	(16)	266	57.5	(50.8	– 64.9)	–	–	–
4 members	270	(21)	403	84.2	(76.2	– 92.8)	–	–	–
5 members	72	(17)	106	68.8	(56.3	– 83.2)	–	–	–
6 members and more	19	(18)	25	61.6	(39.9	– 90.9)	–	–	–
**Member in a household with an income level**									
Low ≤ p30	133	(19)	183	57.5	(49.5	– 66.5)	–	–	–
Medium (p30 – p80)	543	(24)	77	69.9	(65.1	– 74.9)	–	–	–
High ≥p80	848	(33)	1237	84.9	(80.3	– 89.8)	–	–	–

^a^ Number of people who declared being affected by at least one injury

^b^ Number of HLIs adding up all HLIs per person

^c^ Incidence rate per 1000 person-years

^d^ Standardized incidence rate by the France age-sex distribution demographic population estimates in 2015 per 1000 person-years

*Abbreviations*: *HLIs* Home Leisure Injuries, *CIR* Crude Incidence Rate, *SIR* Standardized Incidence Rate, *CI* Confidence Interval

### Injury severity, mechanisms and circumstance of occurrence and consequences

Two out of three HLIs reported in the cohort had no severe consequences, and only 4% required hospitalization (Table 3). The SIR of HLIs requiring medical care was 39.7 HLIs per 1000 person-years (95% CI, 38.1–41.2), 18.0 (95% CI, 17.3–18.8) in men, and 19.1 (95% CI, 18.4–19.9) in women. The age-standardized SIR of HLIs requiring ED attendance or hospitalization was 26.7 HLIs per 1000 person-years (95% CI, 25.6–27.7), 12.6 (95% CI, 12.1–13.0) for men, and 14.1 (95% CI, 13.6–14.7) for women. The CIR of HLIs requiring ED attendance or hospitalization was higher in those under 15 years of age, 50.6 HLIs per 1000 person-years (95% CI, 42.4–60.0), and those of 70 years of age and older, 24.8 HLIs per 1000 person-years (95% CI, 20.2–30.2).

**Table 3:** HLI incidence by type of medical care, mechanism and circumstance of the injury event (activity and location)

HLIs Characteristics	HLIs ^a^ (%)		CIR ^b^ (95% CI)			SIR ^c^ (95% CI)		
**Type of medical care** ^d^ (*n =* 2344)								
No ED attendance or hospitalization	1602	(64)	48.9	(46.5	– 51.3)	53.2	(51.1	– 55.3)
ED attendance	631	(25)	19.3	(17.8	– 20.8)	22.8	(21.9	– 23.7)
Hospitalization	111	(4)	3.4	(2.8	– 4.1)	3.9	(3.7	– 4.0)
**Mechanism** ^d^ (*n =* 2404)								
Struck/hit by fall	1241	(52)	37.9	(35.8	– 40.0)	35.6	(34.2	– 37.0)
Struck/hit by contact with object, person, animal	566	(23)	17.3	(15.9	– 18.8)	18.6	(17.9	– 19.3)
Crushing, cutting, piercing	420	(17)	12.8	(11.6	– 14.1)	11.8	(11.3	– 12.2)
Acute overexertion of body or body part	330	(14)	10.1	(9.0	– 11.2)	9.0	(8.6	– 9.3)
Thermal effect	100	(4)	3.1	(2.5	– 3.7)	3.1	(3.0	– 3.3)
Chemical effect	24	(1)	0.7	(0.5	– 1.1)	1.1	(1.0	– 1.1)
Other	19	(< 1)	0.6	(0.3	– 0.9)	1.6	(1.5	– 1.6)
**Circumstance** (*n =* 2440)								
Sport (without contact with nature)	319	(13)	9.7	(8.7	– 10.9)	15.2	(14.6	– 15.8)
Play and leisure	229	(9)	7.0	(6.1	– 8.0)	11.2	(10.7	– 11.6)
Mobility in public spaces	291	(12)	8.9	(7.9	– 10.0)	8.3	(8.0	– 8.6)
Sport outdoors in contact with nature	230	(9)	9.7	(6.1	– 8.0)	5.9	(5.7	– 6.1)
Vital activities	130	(5)	4.0	(3.3	– 4.7)	5.6	(5.4	– 5.9)
Cooking	152	(6)	4.6	(3.9	– 5.4)	5.0	(4.8	– 5.2)
Walking in the house	151	(6)	4.6	(3.9	– 5.4)	5.2	(5.0	– 5.4)
DIY	216	(9)	6.6	(5.7	– 7.5)	4.7	(4.5	– 4.9)
Climbing stairs in the house	121	(5)	3.7	(3.1	– 4.4)	4.4	(4.2	– 4.6)
Gardening	165	(7)	5.0	(4.3	– 5.9)	3.5	(3.3	– 3.6)
Other domestic activity	104	(4)	3.2	(2.6	– 3.8)	2.8	(2.7	– 2.9)
House cleaning	100	(4)	3.1	(2.5	– 3.7)	2.7	(2.6	– 2.8)
Other mobility	73	(3)	2.2	(1.7	– 2.8)	2.4	(2.3	– 2.5)
Other	126	(5)	3.8	(3.2	– 4.6)	4.5	(4.4	– 4.7)

^a^ Number of HLIs adding up all HLIs per person

^b^ Incidence rate per 1000 person-years

^c^ Standardized incidence rate by the France age-sex distribution demographic population estimates in 2015 per 1000 person-years

^d^ Variable with several possible answers, the percentages of the column do not add up to 100 % of the total number of injuries reported. Events with no data on the type of medical care, mechanism or circumstance, activity or location were not included

*Abbreviations*: *HLIs* Home Leisure Injuries, *CIR* Crude Incidence Rate, *SIR* Standardized Incidence Rate, *CI* Confidence Interval

Despite this overall low level of severity, 73% of the HLIs were reported to be limiting or very limiting for everyday activities in the 48 h following the event (Additional file 2). However, 78% of the HLIs did not result in any work or study stoppage. Among the 14% who lost one or more work/school days, the mean was 22 lost days (Q_1_ = 4 – Q_3_ = 30). The total number of lost work/study days was 613 per 1000 person-years; 38.3% of lost days were due to injuries that did not require ED attendance or hospitalization. We found that most HLI events resulted in single wounds (64%). The most affected body parts were the lower extremities (affected in 52% of the HLIs), the upper extremities (44%), and the head (22%). Contusions (with and without bruising) were the most common typology of injury (32%), followed by abrasion (13%) and sprains (13%). Fractures were the most common typology of injury among hospitalizations. Simple fractures accounted for 35% of hospitalizations, and open fractures for 28% (Additional file 3).

*Struck or hit by falls* was the mechanism with the highest SIR, followed by *struck or hit by contact* with objects, persons, or animals (Table 3). *Struck or hit by falls* was the mechanism that generated the highest number of hospitalizations (73% of HLIs ending with hospitalization). Crushing, cutting or piercing, and overexertion also had high SIR but the severity was generally lower (Additional file 4).

**Fig. 3:**
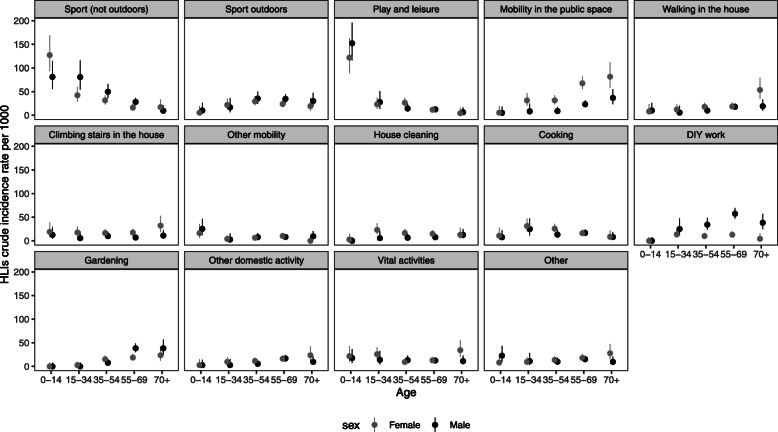
HLI crude incidence rate of HLI circumstances and age-sex groups: crude incidence rate per 1000 person-years, confidence intervals 95%. Events with no data on activity, place, or type of medical care were not included

**Fig. 4:**
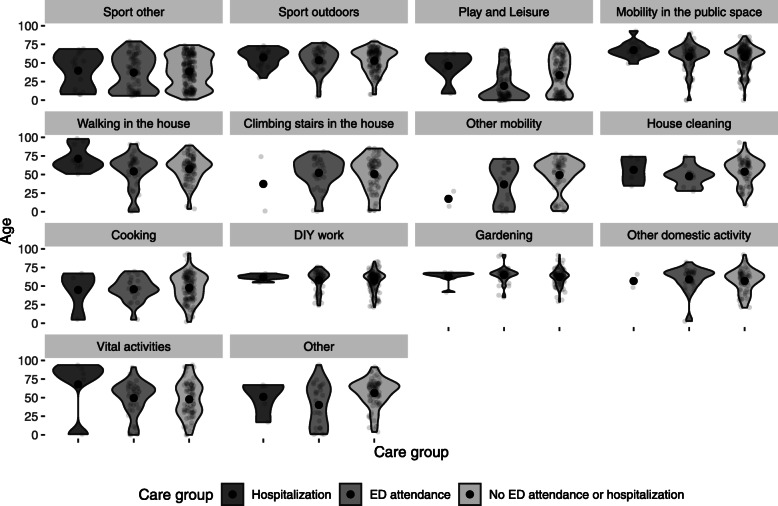
Age of injured people by severity and activity/location groups. The black dots represent the average age. Abbreviation: ED: emergency department. Events with no data on activity, location or type of medical care were not included

The home (50%), the public space (22%), and natural areas (12%) were the most frequent places where HLIs occurred. Walking (20%), sports activities (19%), domestic activities (14%), leisure activities (9%), DIY (9%), and gardening (7%) were the most frequent activities during which HLI occurred (Additional file 5). Table 3 shows the SIRs for each circumstance of occurrence, grouping the most common places and activities of reported HLIs. Figure 3 shows the CIRs for each circumstance of occurrence by age and sex group, and Fig. 4 shows the age distribution of injured participants by HLI severity and circumstances.

Among the circumstances, sports (without contact with nature) top the list, followed by play and leisure. Sports-related injuries affected mainly males, except among children and participants over 70 years old (Fig. 3). Sports, together with playing and leisure, were the circumstances that affected children and young adults the most. Sport, leisure, and playing circumstances frequently involved ED attendance and hospitalizations (36%), mainly among young adults. The highest proportion of HLIs leading to hospitalizations was found among HLIs during outdoor sports activities involving contact with nature (18%). Adults between 55 and 69 years of age were the most affected (67% of hospitalizations by HLIs in sport involving contact with nature). Outdoor sports HLIs occurred mainly while walking or running in environments such as mountains and forests (Additional file 6).

HLIs that occurred while moving represented the second most frequent circumstance (26%), with 12% in public spaces (sidewalks, urban road areas), 6% while walking in the house, 5% while climbing stairs at home, and 3% in other circumstances related to mobility such as riding a bicycle or a scooter. These events primarily affected older adults, most frequently women (Fig. 3). HLIs leading to hospitalizations while moving around (at home or in public places) only concerned participants over 58 years of age.

HLIs were also frequent in the domestic sphere (26%), including do-it-yourself (DIY) activities (9%), gardening (7%), cooking (6%), and house cleaning (4%) (Table 3). DIY activities and gardening showed high SIRs mainly in men, DIY activities primarily in the over-35 age group (with a peak in the 55–69 age group) and gardening in the over-55 age group (Fig. 3). Hospitalizations related to DIY activities, and gardening activities predominantly affected participants around 50 years old. Women were slightly more affected by injuries while cooking or cleaning the house (Fig. 3). These activities presented the lowest frequency of ED attendance or hospitalization (5%).

Other relevant circumstances of HLIs were vital activities (including eating, sleeping, and daily personal hygiene). Both sexes and all ages were affected, but hospitalizations and ED attendances mainly concerned women of 70 years or more (Fig. 3).

## Discussion

The follow-up of participants of the MAVIE cohort led us to estimate an annual SIR of 85.0 HLIs per 1000 person-years (95% CI, 60.6–109.4), measured for 5.2 years (period 2014–2019) and 6302 person-years of follow-up. More than half of HLIs did not require ED attendance or hospitalization. The annual sex-standardized SIR of HLIs was higher in those aged less than 15 and 70 or more. The annual age-standardized SIR of HLIs was higher among women. The CIR of HLIs was higher among women aged 70 or more. The annual SIR of HLIs was higher among participants of high-income households; among *higher managers, participants with professional occupations or independents,* and among retired or pre-retired people; among those living alone and in four-member households. The most common HLI mechanism was the fall (52% of all HLIs, 73% of HLIs ending with hospitalization), while the circumstance of occurrence of HLI that accounted for the highest SIRs of HLIs was sports without contact with nature. The most common circumstance of HLIs leading to hospitalization was outdoor sport activities (in contact with nature) (18%). Conversely, house cleaning and cooking rarely required ED attendance or hospitalization (5%).

The estimated incidences in the MAVIE observatory were lower than those measured in other French studies. The annual SIR of HLIs requiring medical care was 39.7 per 1000 person-years, compared with 178 per 1000 person-years estimated by the ESPS in 2012 (Paget & Thélot, 2017). The annual SIR of HLIs that required ED attendance or hospitalization was 290 per 1000 person-years, compared with 500 per 1000 person-years estimated by the ESPS in 2012 (Paget & Thélot, 2017). The SIR of HLIs that required ED attendance or hospitalization among adults was 21.5 per 1000 person-years, compared with 48.7 per 1000 person-years in the EPAC survey (in the period between 2004 and 2008). A possible explanation for these differences is the underreporting of HLIs and severe HLIs in our sample. Further comparisons with other previous studies are problematic due to differences in designs, outcome definitions and data sources. Some population studies considered only adults (Bonaldi et al., 2014) or mainly men (Verrier & Chevalier, 2007). The HLI definition also varies, including sports injuries (Bonaldi et al., 2014) or not (Verrier & Chevalier, 2007). Finally, the data sources differ, with mortality data (Lasbeur & Thélot, 2014), data obtained from ED attendances (Bonaldi et al., 2014), the combination of ED attendances and medical consultation (Richard et al., 2013), or the combination of ED attendances and outpatient consultation (Paget & Thélot, 2017).

We included HLIs that did not require ED attendance or hospitalization because we considered them to be also relevant. Several studies have shown that injuries of any severity can deteriorate the quality of life and reduce productivity (McClure et al., 2002; Mulder et al., 2002). We found that low-severity HLIs resulted in considerable loss of work or school days. We estimated 109.0 (36.1–301.8) days of work or school lost per 1000 person-years, or about 13.4 million days lost each year in France, in comparison with the 45.9 million days lost in 2019 due to reported work-related injuries (Rapport Annuel 2019, 2019).

In agreement with the literature (Bonaldi et al., 2014), the sex-standardized SIRs of HLIs were higher in children and adolescents and in persons aged 70 years and older. In contrast with the results of other French studies in the general population (Paget & Thélot, 2017; Richard et al., 2013), the age-standardized SIR was higher in women. The largest difference between men and women was found in participants aged 70 years and older, as described for falls (Ek et al., 2019). This difference may be due to the inclusion in our study of low-severity HLIs, often related to domestic activities and recurrent low-severity falls. However, we cannot exclude underreporting of events among men, especially among those aged 70 years and older.

In contrast to other studies that showed that socially disadvantaged people have a higher risk of severe injuries and injuries requiring hospitalization (Ferrante et al., 2014; Cubbin & Smith, 2002), our study showed higher HLI SIRs among household members with higher incomes. This may be related to the grouping of home injuries with sports and leisure injuries in French studies, including ours. The higher participation in sports and leisure activities by members of high-income households may be part of the explanation. In contrast with previous French studies (Paget & Thélot, 2017; Thélot et al., 2017; Dalichampt & Thelot, 2008), we did not find high incidence rates among people with high educational levels, which may be due to the redefinition of the level of education depending on age. The SIRs of HLIs were higher among *higher managers, participants with professional occupations, or independents*. Greater exposure to sports and leisure activities and less experience doing DIY activities may also explain it. The higher SIRs rate of HLIs in retired or pre-retired people is probably related to the higher incidence rates of falls in older people (Peeters et al., 2019) and the exposition to certain risky activities as DIY and gardening. Similar reasons may also explain the higher SIRs rate of HLIs in people living alone, many of whom are older people. Increased SIRs for people living in 4-member households may be related to the presence of children at home, as shown by a recent study of home injuries in the United States (Gielen et al., 2020).

As already suggested by other studies (Verrier & Chevalier, 2007; EPAC, 2017; INVS, 2017), our study shows that incidence, causes, and consequences of HLIs differ by age group and sex, and are related to the performance of different activities. During the study of the circumstances of occurrence of HLIs, patterns of occurrence have emerged: playing games and playing sports in children; playing sports among adolescents, young adults and particularly among men; household activities (cooking, cleaning, etc.) among middle-aged adults and older adults, especially women; DIY and gardening among men, middle-aged adults and older adults, especially older men; mobility (in the public space, at home, going up and downstairs) in women, especially older women; and life activities in older adults.

A high incidence rate of sport-related HLIs in the MAVIE cohort was expected, as the practice of sports in France is frequent, especially outdoor sports. According to a survey on sports practices conducted in 2018 by the Ministry of Sports (INSEP, 2010), two-thirds of French people aged 15 years and older reported doing physical activity at least once a year, and only 18% reported never having practiced sports or physical activity. Among the French adults, 85% of men and 76% of women between 15 and 74 years of age reported practicing sports other than leisure and utility walking. The percentages were 46% for men and 34% for women aged 75 and over (INSEP, 2010).

In our study, we identified nature as a place where severe HLIs occurred frequently. HLIs leading to hospitalization occurred most frequently during the practice of outdoor sports activities (in contact with nature) (18%).

Another finding of our study was the higher proportion of severe HLIs in persons aged 50 to 70 years (48% of all severe HLIs that required hospitalization). Sports, including outdoor sports, gardening, and housework, were frequent circumstances of the most severe HLIs. Hospitalizations for mobility-related HLIs (at home or in public places) only affected persons older than 58. This result is consistent with the known importance of falls in the elderly.

### Strengths and limitations

The main strengths of the MAVIE observatory are associated with the web-based management and the prospective design of the cohort (Rojas-Castro et al., 2021). Web-based management simplified logistics (invitations, registration, data collection, and follow-up), reduced recruitment costs and facilitated the implementation of different strategies to attract the attention or retention of participants. The prospective design contributed to reducing recall bias and ensured that the exposures preceded the HLI events (Rojas-Castro et al., 2021).

Our study had limitations common to other volunteer-based cohorts and e-cohorts. The response rate was low; the analyses included 8638 people, while almost 4 million e-mail invitations had been sent. The follow-up rate (86.3%) was higher than the 60–80% range considered acceptable by some authors (Kristman et al., 2004). The MAVIE cohort differs demographically from the French population: children, adolescents, young adults, and participants aged 75 years or older are underrepresented. Participants between 50 and 70 years of age, and participants of high socioeconomic and educational level are overrepresented. Previous research summarizes our efforts to deal with representativeness, loss of follow-up, and information quality (Rojas-Castro et al., 2021).

The underrepresentation of young people may have led to underestimating the incidences of sports and leisure time injuries. The underrepresentation of persons older than 75 years may have led to an underestimation of the incidences of falls while moving. The possible overrepresentation of participants with a history of HLIs (because a recent HLI in the family or friends circle would have motivated them to participate) may have led to an overestimation of the incidence of HLIs. We discarded events that occurred just at entry into the cohort (264 events in total) to reduce this possible bias.

The lack of representativeness implies that incidence estimates should not be generalized and should be used exclusively to compare subgroups and study risk factors (Richiardi et al., 2013). The generalization of these results to the French population should be made with caution. Methods such as calibration, stratification and imputation could help correct the lack of representativeness (Keiding & Louis, 2017) in future studies. The following steps of the research are the study of risk factors for HLIs, for which the sample is not required to be representative of the general population (Keiding & Louis, 2017). We intend to study the intrinsic and environmental risk factors for each type of HLIs, when the sample size and number of events allow it.

## Conclusion

Despite the limitations, the present study provides a broad picture of the public health burden of the main types of HLI in the French population, particularly those of minor and moderate severity. We show that HLIs differ qualitatively and quantitatively by age group. Emerging patterns of occurrence are mainly related to sports and leisure, mobility, DIY work, domestic and gardening activities. Considering that these patterns are the product of the risk of a given activity, the time spent on the activity (exposure time), and possible individual vulnerabilities, further analyses should focus on disentangling these factors. Our results support the utility of prevention interventions, policies, and campaigns targeting sub-populations and specific types of HLIs. Understanding which modifiable factors can reduce risk by age group and specific activities is essential for the success of these programs.

## Supplementary Information

**Additional file 1.** Percentage of practitioners of different domestic, sport and leisure activities among volunteers of the cohort MAVIE.

**Additional file 2.** Consequences of HLIs by type of medical care. Events with no data on the consequences of the injury were not included.

**Additional file 3.** Typology of injury by type of medical care.

**Additional file 4.** Mechanisms of HLIs by type of medical care.

**Additional file 5.** Activities and locations where volunteers suffered the most HLIs. Mosaic graphic resenting activity and location of the accident. Surface areas are proportional to the number of reported injuries. Colours refer to locations where accidents occurred.

**Additional file 6.** Activities and detail locations where volunteers suffered the most HLIs. Mosaic graphic resenting activity and detail location of the accident. Surface areas are proportional to the number of reported injuries. Colours refer to general locations where accidents occurred.

## Data Availability

Personal health data underlying the study are protected by the French Data Protection Act and cannot be shared publicly. The large number of variables allows data to be indirectly identifiable and making such data freely available is prohibited. Furthermore, an authorization from the *Commission Nationale de l’Informatique et des Libertés*, the French Data Protection Authority, may be required to transfer the data, especially abroad. The MAVIE observatory protocol was approved by the *Commission Nationale de l’Informatique et des Libertés* on May 28, 2013 (decision DR-2013-288). Data from this study can be obtained upon request from the steering committee (http://www.observatoire-mavie.com/contacter-equipe-MAVIE.aspx), as well as from the corresponding author (emmanuel.lagarde@u-bordeaux.fr).

## Abbreviations

CIR: Crude Incidence Rate
DIY: Do-It-Yourself
ED: Emergency Department
EPAC: The French home and leisure injury permanent survey (translated from French)
ESPS: The Health, health care and insurance survey (translated from French)
EU: European Union
HLI: Home and Leisure Injury
MAVIE: Mutualists against Home and Leisure Injuries (translated from French)
SIR: Standardized Incidence Rate
PTFR: Person-Time Follow-up Rate
