# Supplementary figures and images for: A web-based prospective cohort study of home, leisure, school and sports injuries in France: a descriptive analysis
Source: Inj Epidemiol. 2021 Aug 4;8:50. doi: 10.1186/s40621-021-00343-9 (PMC8336358; doi:10.1186/s40621-021-00343-9)

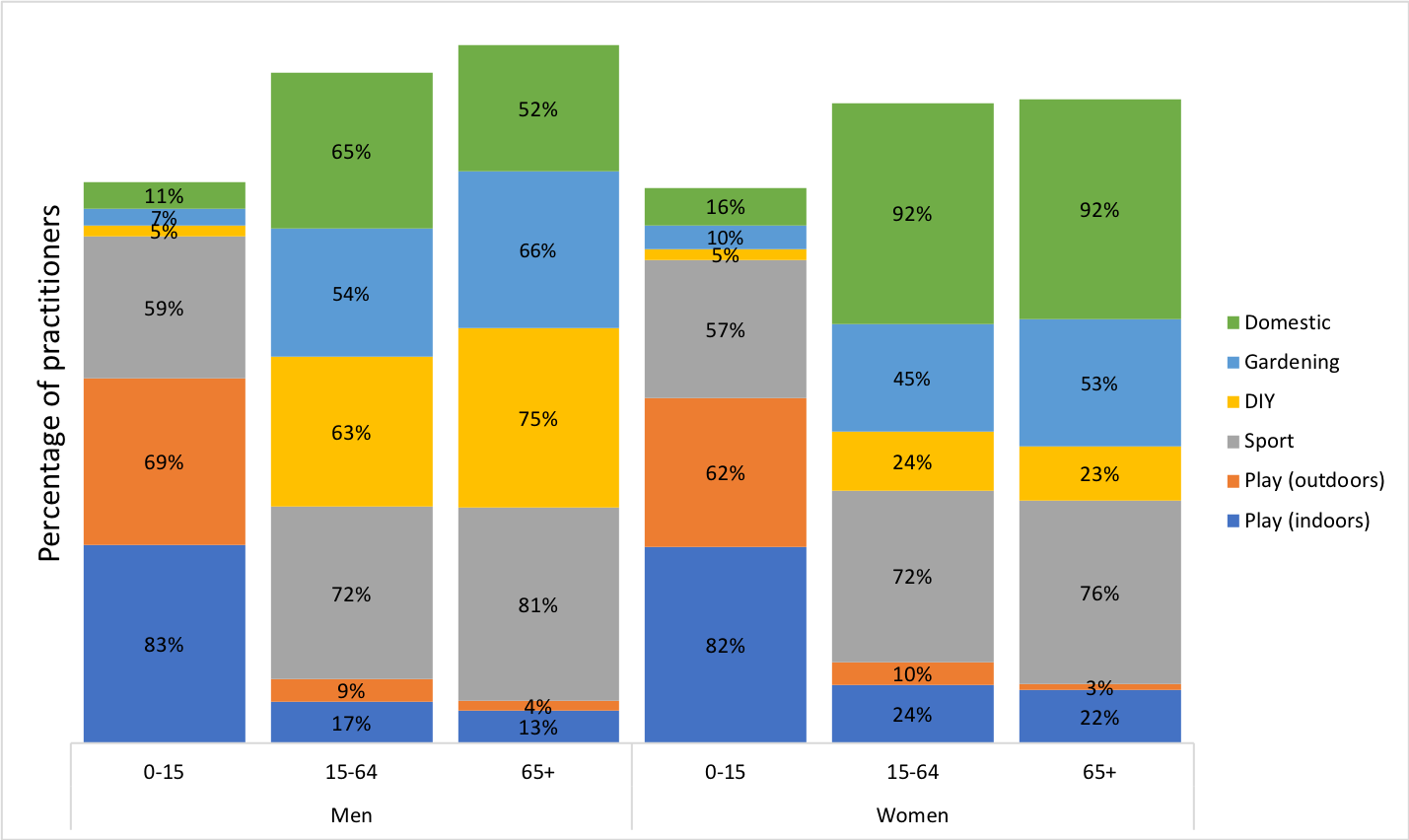

Supplement: Supplementary file 1 — Additional file 1. Percentage of practitioners of different domestic, sport and leisure activities among volunteers of the cohort MAVIE. [file 40621_2021_343_MOESM1_ESM.png]

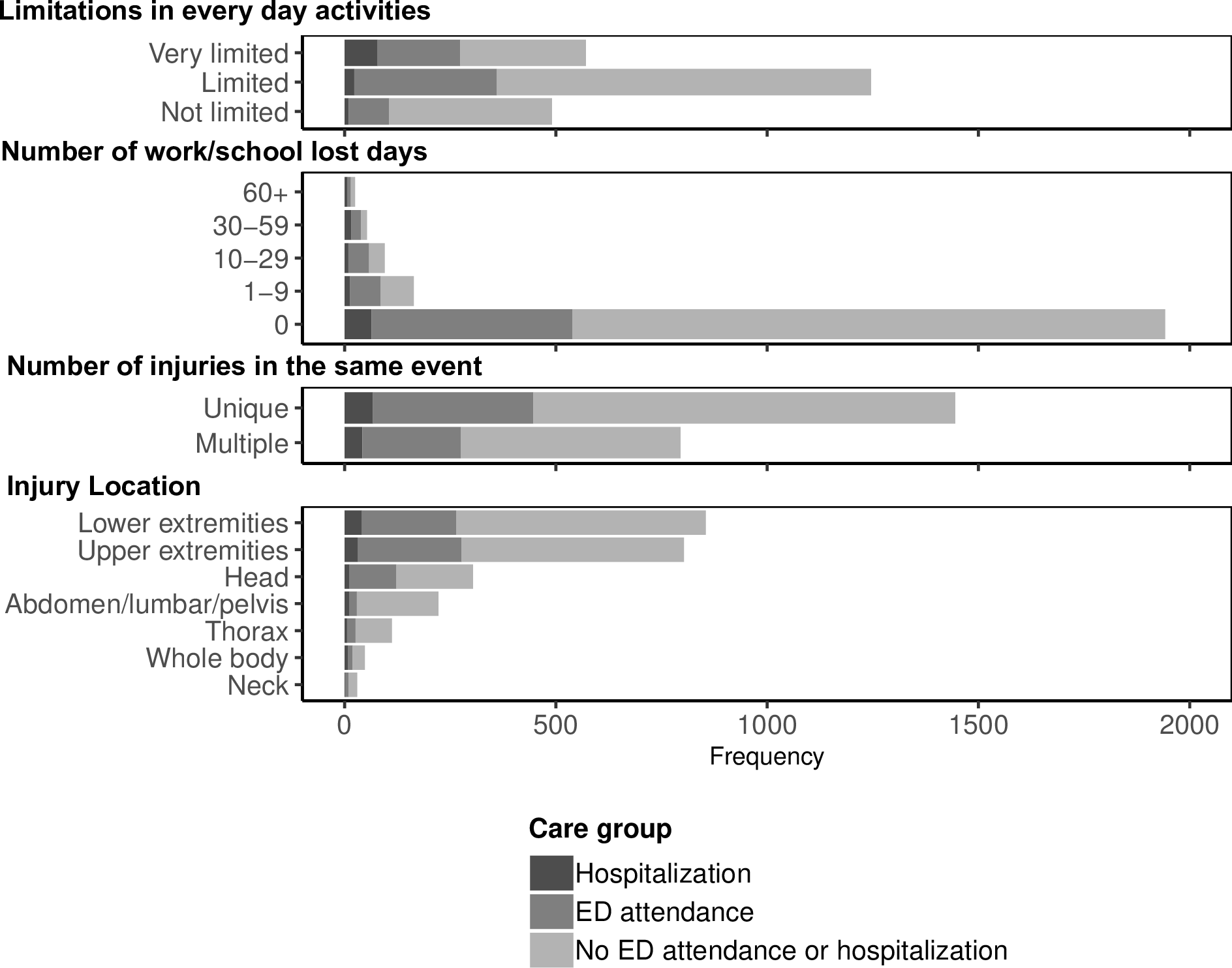

Supplement: Supplementary file 2 — Additional file 2. Consequences of HLIs by type of medical care. Events with no data on the consequences of the injury were not included. [file 40621_2021_343_MOESM2_ESM.tif]

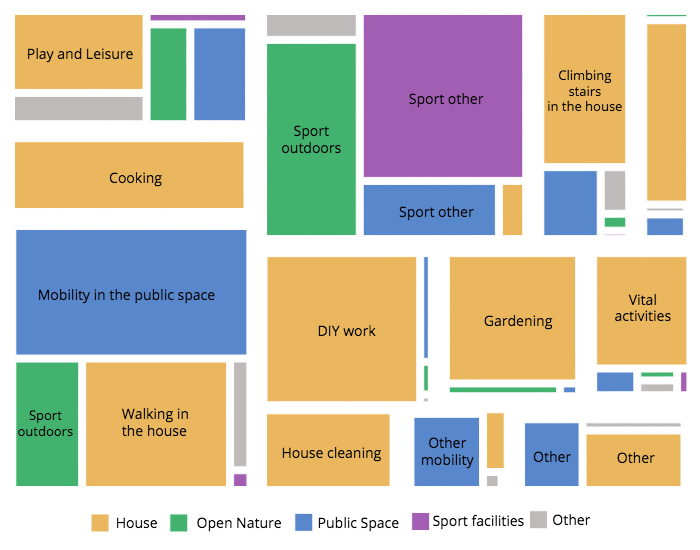

Supplement: Supplementary file 5 — Additional file 5. Activities and locations where volunteers suffered the most HLIs. Mosaic graphic resenting activity and location of the accident. Surface areas are proportional to the number of reported injuries. Colours refer to locations where accidents occurred. [file 40621_2021_343_MOESM5_ESM.jpg]

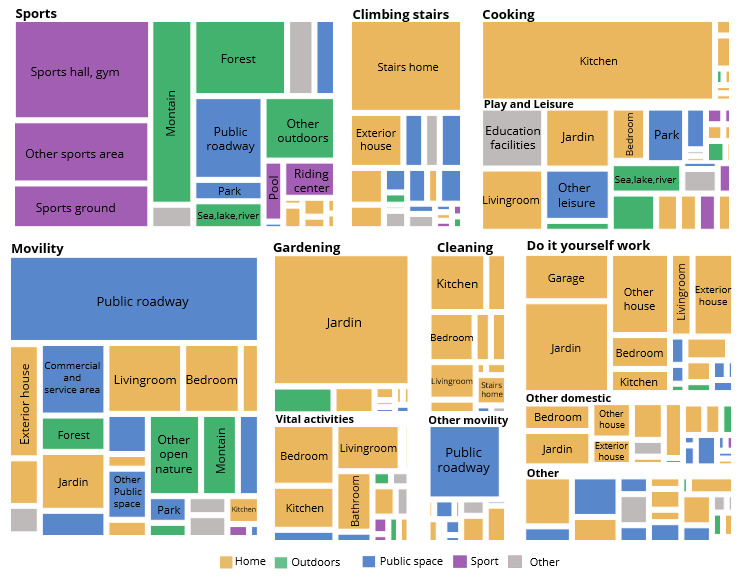

Supplement: Supplementary file 6 — Additional file 6. Activities and detail locations where volunteers suffered the most HLIs. Mosaic graphic resenting activity and detail location of the accident. Surface areas are proportional to the number of reported injuries. Colours refer to general locations where accidents occurred. [file 40621_2021_343_MOESM6_ESM.jpg]
