# Supplementary material for: A web-based prospective cohort study of home, leisure, school and sports injuries in France: a descriptive analysis
Source: Inj Epidemiol. 2021 Aug 4;8:50. doi: 10.1186/s40621-021-00343-9 (PMC8336358; doi:10.1186/s40621-021-00343-9)
Supplement: Supplementary file 3 — Additional file 3. Typology of injury by type of medical care. [file 40621_2021_343_MOESM3_ESM.docx]

**Additional file 2– Typology of injury by the type of medical care.**

| **HLIs Type*** | **Hospitalization (%)** | | **ED attendance (%)** | | **No ED attendance or Hospitalization (%)** | |
| --- | --- | --- | --- | --- | --- | --- |
| Simple fracture | 39 | (35.1) | 154 | (24.4) | 127 | (7.9) |
| Open fracture | 31 | (27.9) | 22 | (3.5) | 7 | (<1) |
| Contusion/bruise | 21 | (18.9) | 213 | (33.8) | 558 | (34.8) |
| Luxation/dislocation | 12 | (10.8) | 28 | (4.4) | 45 | (2.8) |
| Abrasion/scratch | 7 | (6.3) | 73 | (11.6) | 250 | (15.6) |
| Tendon lesion | 6 | (5.4) | 27 | (4.3) | 86 | (5.4) |
| No diagnosed | 5 | (4.5) | 2 | (0.3) | 52 | (3.2) |
| Crushing | 4 | (3.6) | 24 | (3.8) | 62 | (3.9) |
| Lesion of blood vessel | 4 | (3.6) | 14 | (2.2) | 33 | (2.1) |
| Meniscus cartilage lesion | 3 | (2.7) | 13 | (2.1) | 32 | (2.0) |
| Severe muscular lesion | 2 | (1.8) | 7 | (1.1) | 40 | (2.5) |
| Severe sprain/ligament rupture | 2 | (1.8) | 39 | (6.2) | 29 | (1.8) |
| Slight sprain/distortion | 2 | (1.8) | 98 | (15.5) | 225 | (14.0) |
| Light muscle lesion | 1 | (<1) | 22 | (3.5) | 163 | (10.2) |
| Skin damage | 1 | (<1) | 15 | (2.4) | 80 | (5.0) |
| Slight ligament lesion | - | (<1) | 18 | (2.9) | 70 | (4.4) |
| Thermal burn | - | (<1) | 11 | (1.7) | 76 | (4.7) |
| Hypothermia | - | (<1) | - | (<1) | 1 | (<1) |
| Other | 18 | (16.2) | 9 | (10.9) | 184 | (11.5) |

*Variable with several possible answers, the percentages of the columns do not add up to one hundred. This table includes categories that added up to at least one percent of the observed events. Events with no data on the typology of injury were not included
