# Supplementary material for: A web-based prospective cohort study of home, leisure, school and sports injuries in France: a descriptive analysis
Source: Inj Epidemiol. 2021 Aug 4;8:50. doi: 10.1186/s40621-021-00343-9 (PMC8336358; doi:10.1186/s40621-021-00343-9)
Supplement: Supplementary file 4 — Additional file 4. Mechanisms of HLIs by type of medical care. [file 40621_2021_343_MOESM4_ESM.docx]

**Additional file 4**–**Mechanisms of HLIs by type of medical care**

| **Mechanisms*** | **Hospitalization (%)** | | **ED attendance (%)** | | **No ED attendance or hospitalization (%)** | |
| --- | --- | --- | --- | --- | --- | --- |
| Struck/hit by fall | 81 | (73) | 352 | (56) | 762 | (48) |
| Struck/hit by contact with object, person, animal | 21 | (19) | 196 | (31) | 332 | (21) |
| Acute overexertion of body or  body part | 9 | (8) | 42 | (7) | 273 | (17) |
| Crushing, cutting, piercing | 8 | (7) | 136 | (22) | 269 | (17) |
| Chemical effect | 6 | (5) | 3 | (<1) | 14 | (<1) |
| Thermal effect | - | (<1) | 9 | (1) | 91 | (6) |
| Other | 3 | (3) | 2 | (<1) | 10 | (<1) |

*Variable with several possible answers, the percentages of the columns do not add up to one hundred percent of the total number of injuries reported. Events with no data on mechanisms were not included
